# Bringing data to life: A model for involving lived experience in data-driven research

**DOI:** 10.23889/ijpds.v9i5.2499

**Published:** 2024-09-18

**Authors:** Sarah McKenna, Elizabeth Nelson, Aideen Maguire

**Affiliations:** 1 Queen's University Belfast; 2 Ulster University

## Objective

It is generally accepted that involving experts by experience is necessary to deliver research that is relevant to community need and to progress the data democratisation agenda. We have piloted and evaluated a model of co-production for data-driven research with seldom heard groups.

## Approach

In partnership with the charity Voice of Young People in Care (VOYPIC) and a group of care experienced young people, the Administrative Data Research Centre Northern Ireland (ADRC NI) piloted and implemented a model of co-production in data-driven research. To evaluate the model, interviews were conducted with the young people, staff at VOYPIC, and ADRC NI researchers and public engagement team members.

## Results

Thematic analysis of interview data identified numerous benefits and barriers to co-producing data-driven research with experts by experience. We offer reflections from three perspectives (the young people, VOYPIC and ADRC NI), as well as a model that can be adapted with different lived experience groups in other data-driven research programmes.

## Conclusion

Experts by experience can be equal partners in most stages of the data research cycle, and there are tangible benefits for all partners and the research itself. However, aligning the aspirational principles of co-production with the realities of delivering data research is challenging and has implications for achieving the democratisation of data.

## Conclusion

This session will share real world perspectives on the co-production of data research with seldom heard groups.

